# Transmission of SARS-CoV-2 in the Population Living in High- and Low-Density Gradient Areas in Dhaka, Bangladesh

**DOI:** 10.3390/tropicalmed7040053

**Published:** 2022-03-25

**Authors:** Syed Moinuddin Satter, Taufiqur Rahman Bhuiyan, Zarin Abdullah, Marjahan Akhtar, Aklima Akter, S. M. Zafor Shafique, Muhammad Rashedul Alam, Kamal Ibne Amin Chowdhury, Arifa Nazneen, Nadia Ali Rimi, A. S. M. Alamgir, Mahbubur Rahman, Farzana Islam Khan, Tahmina Shirin, Meerjady Sabrina Flora, Sayera Banu, Mustafizur Rahman, Mahmudur Rahman, Firdausi Qadri

**Affiliations:** 1 Programme for Emerging Infections, Infectious Diseases Division, icddr,b, Dhaka 1212, Bangladesh; taufiqur@icddrb.org (T.R.B.); zarin.abdullah@icddrb.org (Z.A.); marjahan.akhtar@icddrb.org (M.A.); aklima17@gmail.com (A.A.); zafor.shafique@icddrb.org (S.M.Z.S.); rashedul.alam@icddrb.org (M.R.A.); kiachowdhury@icddrb.org (K.I.A.C.); arifa.nazneen@icddrb.org (A.N.); nadiarimi@icddrb.org (N.A.R.); sbanu@icddrb.org (S.B.); mustafizur@icddrb.org (M.R.); fqadri@icddrb.org (F.Q.); 2 Institute of Epidemiology, Disease Control & Research, 44 Mohakhali, Dhaka 1212, Bangladesh; dr.alamgir@iedcr.gov.bd (A.S.M.A.); dr.mahbub@iedcr.gov.bd (M.R.); dr.farzana@iedcr.gov.bd (F.I.K.); director@iedcr.gov.bd (T.S.); 3 Directorate General of Health Services (DGHS), Mohakhali, Dhaka 1212, Bangladesh; adgplanning@ld.dghs.gov.bd; 4 Global Health Development, EMPHNET, 69 Mohakhali, Dhaka 1212, Bangladesh; mrahman@globalhealthdev.org

**Keywords:** COVID-19, SARS-CoV-2, community transmission, population density gradient, Dhaka, Bangladesh

## Abstract

Community transmission of SARS-CoV-2 in densely populated countries has been a topic of concern from the beginning of the pandemic. Evidence of community transmission of SARS-CoV-2 according to population density gradient and socio-economic status (SES) is limited. In June–September 2020, we conducted a descriptive longitudinal study to determine the community transmission of SARS-CoV-2 in high- and low-density areas in Dhaka city. The Secondary Attack Rate (SAR) was 10% in high-density areas compared to 20% in low-density areas. People with high SES had a significantly higher level of SARS-CoV-2-specific Immunoglobulin G (IgG) antibodies on study days 1 (*p* = 0.01) and 28 (*p* = 0.03) compared to those with low SES in high-density areas. In contrast, the levels of seropositivity of SARS-CoV-2-specific Immunoglobulin M (IgM) were comparable (*p* > 0.05) in people with high and low SES on both study days 1 and 28 in both high- and low-density areas. Due to the similar household size, no differences in the seropositivity rates depending on the population gradient were observed. However, people with high SES showed higher seroconversion rates compared to people with low SES. As no difference was observed based on population density, the SES might play a role in SARS-CoV-2 transmission, an issue that calls for further in-depth studies to better understand the community transmission of SARS-CoV-2.

## 1. Introduction

The COVID-19 pandemic, caused by the novel severe acute respiratory syndrome coronavirus 2 (SARS-CoV-2), has affected 450 million people, with 6.01 million deaths, worldwide up to 8 March 2022. The transmission of SARS-CoV-2 from an index case has been documented to occur following close contact through infected secretions such as saliva and respiratory secretions or respiratory droplets, as well as other body fluids [1,2]. Secondary attack rates, which indicate how interactions relate to the transmission risk, have been estimated at 3.3% for SARS-CoV-2, 16.1% of which following household contacts, and 1.1% following social contacts [3]. The basic reproduction number (R0) of SARS-CoV-2, an indication of the virus’s initial transmissibility, was estimated to be 4.71 (range of 4.50–4.92) when the pandemic started in December 2019 [4]. In recent publications, the basic reproduction numbers of SARS-CoV-2 were observed to vary in the range of 1.0011–2.7936 for different countries [5]. Worldwide, the parameters of transmission dynamics of SARS-CoV-2 have been estimated mostly among household or social contacts. However, evidence on transmission dynamics of SARS-CoV-2 according to population density gradients in low- and middle-income countries was scarce when this study was started. Bangladesh is a densely populated country, with 1116 people living per square kilometer, and in Dhaka, the capital, it is estimated that 220,246 persons live per square kilometer (km) in high-density areas like slums [3], and 29,857 persons live per square kilometer (km) in low-density areas such as non-slums [6]. On 8 March 2020, the Government of Bangladesh reported the first case of SARS-CoV-2, and as of May 2021, close to a million people have tested positive for SARS-CoV-2 in Bangladesh, with over 12,549 confirmed deaths [7]. From the data of the Bangladesh Bureau of Statistics, it has been observed that there are significant differences in population density in different areas of Dhaka city. Therefore, we assumed that the transmission dynamics of SARS-CoV-2 might be diverse according to population density gradients. Moreover, people in Bangladesh mostly maintain a robust social network, and community members interact with each other often. This practice might also contribute to the community transmission of SARS-CoV-2 but may differ according to the population density. From mid-April 2020 up to December 2020, a nationwide community-based transmission study on “Transmission Dynamics of COVID-19 in Bangladesh” was carried out both in rural and urban areas to estimate the secondary attack rate (SAR) and the basic reproduction number (R0) among household contacts. At that time, we were not aware that the parameters of transmission dynamics of SARS-CoV-2 may differ among contacts of SARS-CoV-2 index cases according to the population density gradient of Dhaka city. Therefore, we initiated this study intending to estimate the secondary attack rate (SAR) and basic reproduction number (R0) among contacts in high- and low-density areas of Dhaka city. Our hypothesis was that SAR would be higher in high-density areas because of the local social structure and behavior patterns. For a long time, the Government of Bangladesh implemented area-wise lock-down or mobility restrictions depending upon the level of risk of infection in different communities. The findings of this study aim to supplement governmental policies for future outbreaks of SARS-CoV-2. To gain a comprehensive understanding of the susceptible population, we also tested for sero-positivity people who reported household or neighborhood contacts with a laboratory-confirmed case. We also collected qualitative data on risk perception and prevention practices such as masking and social distancing in high- and low-density populations in Bangladesh, which will be reported in a subsequent article. Here, we report key epidemiological and laboratory-based data from a longitudinal study of SARS-CoV-2 transmission among household and neighborhood contacts.

## 2. Materials and Methods

### 2.1. Study Design and Settings

The study design was longitudinal, and its duration was 6 months, commencing on 27 June 2020. In the beginning, we located laboratory-confirmed index cases in high-density communities of six slums and low-density communities of seven wards of Dhaka city through the “Transmission Dynamics of COVID-19 in Bangladesh” study. The detailed methodology of symptomatic and asymptomatic index case enrollment was described elsewhere [8]. Then, the cases were interviewed to trace their home and neighborhood contacts. We followed World Health Organization (WHO) contact definition considering our study and country context. We considered an individual as a contact who experienced any of the following exposures during the 2 days before and the 14 days after the onset of symptoms of a laboratory-confirmed COVID-19 case: (1) face-to-face contact with a confirmed case within 1 m and for more than 15 min (including travel, gossips, tea stall) or (2) direct physical contact with a confirmed COVID-19 case. The contacts were communicated by the team for verification of the exposure to the case and possible enrollment. After enrollment, collection of epidemiological data and specimens was done. Nasopharyngeal samples were collected on day 1, day 7, day 14, and day 28 for RT-PCR. Blood samples were collected on day 1 and day 28 for ELISA antibody test for seropositivity. The contacts were followed up for 14 days for signs and symptoms. The distance from the index case household to their neighbor was determined using a Global Positioning System (GPS) tracker.

### 2.2. Participants and Procedures

We used operational definitions to describe high- and low-density neighborhoods (see Appendix A). In high-density neighborhoods, considering an overall SAR at a neighborhood of 20% with a 95% confidence interval, a 5% desired precision, and a 1.5 design (household cluster) effect, it was estimated that 365 exposed contacts were required [9]. After considering a 10% loss to follow-up and a 15% non-response rate (refusal/non-availability), an estimated 460 neighborhood contacts had to be enrolled. In low-density neighborhoods, a similar methodology was followed, with an overall SAR per neighborhood of 5%; we estimated that 143 contacts needed to be enrolled. We assumed that one case would yield 15–20 contacts [10]. We estimated to approach 31 cases in high-density neighborhoods and 10 cases in low-density neighborhoods to enroll the estimated number of contacts. However, during the fieldwork, we stopped after the enrollment of the 14th index case, as we reached the target number of contacts (*n* = 460). On the other hand, we had to enroll more cases (*n* = 23) to reach the estimated target number of controls (*n* = 143).

### 2.3. Laboratory Testing

#### 2.3.1. SARS-CoV-2 RT-PCR

Viral RNA was extracted and purified from nasopharyngeal swab samples using the Invimag Pathogen kit and an automatic extractor (KingFisher Flex96 system). SARS-CoV-2 detection was performed using a semi-quantitative, matrix gene-specific, probe-based real-time reverse-transcription polymerase chain reaction (RT-qPCR) assay.

#### 2.3.2. SARS-CoV-2-Specific Enzyme-Linked Immunosorbent Assay (ELISA)

The Receptor Binding Domain (RBD) of the spike protein of SARS-CoV-2 was used as an antigen to detect antibody responses as discussed previously (Akter et al., 2021, manuscript in review). RBD-specific IgG and IgM antibody responses were measured using a monoclonal antibody (CR3022) of known concentration, specific to SARS-CoV-2 RBD. This ELISA procedure was validated and described previously [11] (Akter et al., 2021, manuscript in review). Using serum from pre-pandemic healthy controls, we determined the concentration of 500 ng/mL (0.5 µg/mL) as a cut-off value for seropositivity for both RBD-specific IgG and IgM antibodies.

### 2.4. Statistical Analysis

We summarized all categorical variables using frequency and percentage, and all symmetric continuous variables using mean and standard deviation. All variables not having a normal distribution are presented using a median and inter-quartile range. The results from the seroprevalence data were used for the calculation of the fraction of the population that was susceptible.

The secondary attack rate was calculated by dividing the number of positive SARS-CoV-2 contacts on any day of sample collection by the number of contacts enrolled and is presented as a proportion. The basic reproduction number was calculated by dividing the positive SARS-CoV-2 contacts during 14 days of follow-up by the number of index cases. χ^2^ tests were used to compare proportions, and Wilcoxon rank-sum test was used for continuous variables.

We analyzed seroprevalence data based on socioeconomic status in high- and low-density areas as we did not observe any difference in the seroprevalence level depending on the density gradient. Statistical differences in the antibody levels between high- and low-SES groups were analyzed using the Mann–Whitney U test. *p*-values < 0.05 were considered statistically significant.

Written informed consent was obtained from the enrolled cases and contacts. The study protocol was reviewed and approved by icddr,b’s Research Review and Ethical Review Committees.

## 3. Results

### 3.1. Epidemiological Findings

**Figure 1:**
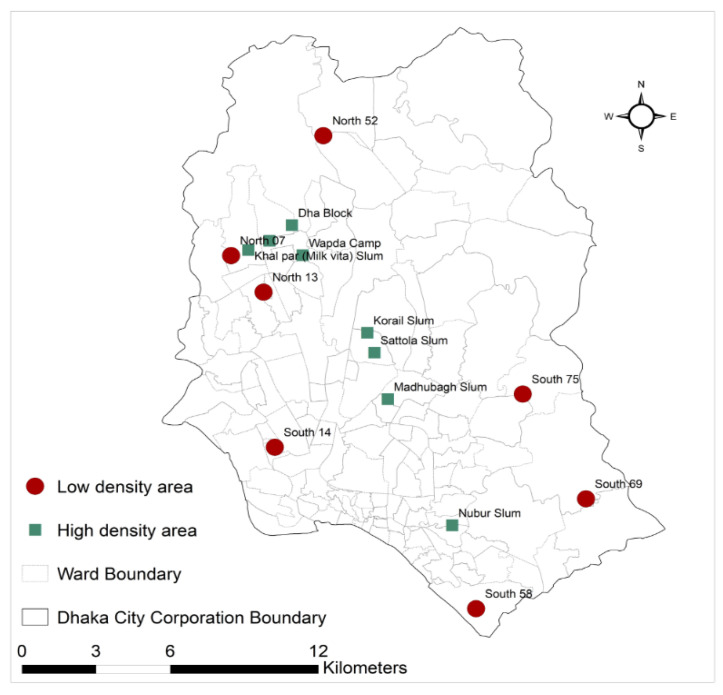
Map showing the location of the selected high- and low-density areas of Dhaka city.

From 27 June 2020 to 26 September 2020, 14 and 23 index cases were enrolled from high- and low-density areas, respectively. During this period, 497 contacts were enrolled from high-density areas, and 187 contacts from low-density areas. The average number of contacts per case was 36 in high-density areas and 8 in low-density areas (Figure 1).

The total number of refusals was 107 in high-density areas and 40 in low-density areas. The primary reasons for not being able to collect the samples were absence from home (47%), refusal (38%), and migration (15%). Most of the enrolled contacts were in the 11–30 age groups in high- and low-density areas (Table 1). Enrollment of female contacts was higher in both high- (54%) and low-density (53%) areas (Table 1). The contacts had mostly a primary education level in both high- (40%) and low-density (52%) areas (Table 1). Ten percent of the contacts (10%, 50/497) were SARS-CoV-2-positive in high-density areas, and 20% (37/187) were SARS-CoV-2-positive in low-density areas. SARS-CoV-2 was identified at least in one of four nasopharyngeal specimens, collected on days 1, 7, 14, and 28.

In high-density areas, 91% (452/497) of the contacts were asymptomatic during enrollment, compared to 75% (141/187) in low-density areas. Among them, 7% (31/452) developed symptoms within 14 days of follow-up, and 13% (4/31) were diagnosed as SARS-CoV-2-positive in high-density areas. Eleven percent (16/141) of the contacts later developed symptoms in low-density areas, and 31% (5/16) became SARS-CoV-2-positive. The detection of new positive contacts was highest on day 1 in both high-density (48%) and low-density (54%) areas compared to the follow-up days at 7, 14, and 28.

The highest proportions of SARS-CoV-2-positive subjects were detected among contacts aged between 21 and 30 years in both high- (30%) and low-density (46%) areas. In high-density areas, 40% of males were infected with SARS-CoV-2, whereas in low-density areas, 32% of males were infected with SARS-CoV-2.

Overall, the secondary attack rate (SAR) was 13% (87/684), and the SAR among contacts was 10% in high-density areas compared to 20% in low-density areas (Table 2). The basic reproduction number (R0) was 2.7 in high-density areas and 1 in low-density areas (Table 3).

**Figure A1:**
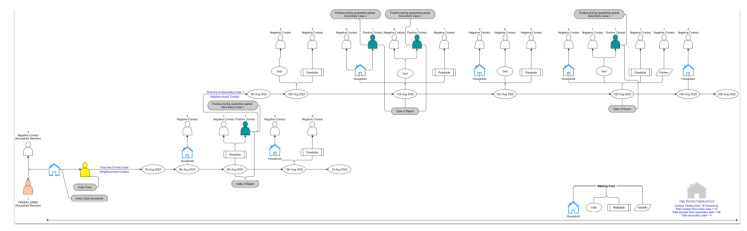
Diagram showing the setting of exposure and the direction of transmission from index case to household and neighborhood contacts in high-density areas in Dhaka city.

**Figure A2:**
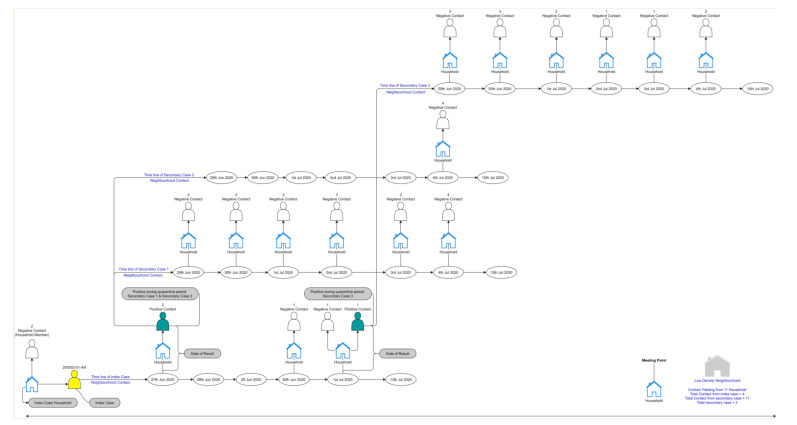
Diagram showing the setting of exposure and the direction of transmission from index case to household and neighborhood contacts in low-density areas in Dhaka city.

The effective reproduction number was higher than 1 in high-density areas (1.4), whereas it was lower than 1 in low-density areas (0.71). By using a GPS tracker, a total of 497 contacts from 268 households in high-density areas and of 187 contacts from 92 households in low-density areas were identified. We observed that the average distance between an index case household and a contact household was 35 m in high-density areas, whereas it was 44 m in low-density areas. We found positive contacts up to a distance of 250 m from an index case household in high-density areas and of up to 440 m away from an index case household in low-density areas (Figure A1 and Figure A2).

### 3.2. SARS-CoV-2-Specific Antibody Responses in Relation to High and Low SES

Primarily, we analyzed the seroprevalence of SARS-CoV-2 antibodies in high- and low-density areas of Dhaka city. However, there was no difference in the magnitude and frequencies (*p* > 0.05; data not shown) in the level of SARS-CoV-2 antibodies between people in high- and low-density areas of Dhaka. Thereafter, we performed additional seroprevalence analyses for SARS-CoV-2 antibodies comparing people with high and low socioeconomic status living within high- and low-density areas. We determined SARS-CoV-2-specific IgG and IgM seropositivity in all individuals on study day 1 and day 28. People living in high-density areas with high SES had significantly higher levels of SARS-CoV-2-specific IgG antibodies on both study day 1 (*p* = 0.011) and study day 28 (*p* = 0.005) compared to the people with low SES. In contrast, this effect was not observed in the low-density areas (Table 4). IgG seropositivity was also significantly higher in high-SES people living in high-density areas than in low-SES participants on both study day 1 (73% vs. 59%, *p* = 0.011) and study day 28 (74% vs. 59%, *p* = 0.005). In contrast, the level of seropositivity for SARS-CoV-2-specific IgM was comparable (*p* > 0.05) in people with high and low socioeconomic status on both study day 1 and study day 28 (Table 4).

Next, we analyzed seropositivity among RT-PCR-positive contacts found on days 1, 7, 14, and 28. Overall, individuals with both low and high SES who were RT-PCR-positive on day 1 had increased levels of IgG antibodies on day 28 (Table A1). IgG seropositivity also rose from 62–69% (day 1) to 100% (day 28), but no statistically significant differences were observed between high- and low-SES participants. In contrast, when considering the RT-PCR-positive individuals on day 7, high-SES participants had significantly higher (*p* < 0.005) seropositivity on day 1 compared to low-SES individuals (70% vs. 45%). A similar trend was observed among high-SES individuals who were RT-PCR-positive on study days 14 and 28 (Table A1).

No apparent increase in IgM seropositivity was observed between study day 1 and day 28 among RT-PCR-positive individuals. Higher, but not significant, IgM antibody levels were found on day 28 in RT-PCR-positive participants compared to day 1. Among RT-PCR-positive individuals on day 14, high-SES participants had significantly higher (*p* = 0.006) seropositivity on day 1 compared to low-SES subjects (67% vs. 47%) (Table A1).

## 4. Discussion

To our knowledge, this is the first population-based study on secondary attack rate (SAR) and prevalence of antibodies in people affected by COVID-19 in Bangladesh as well as in South East Asia. In Bangladesh, particularly, a second wave of the pandemic started just a year after the first case was detected in In March 2020. Interestingly, in our study, we did not observe any difference in the frequencies of SARS-CoV-2-specific antibodies in people living in areas with different density gradients in Dhaka city. Since we followed up active cases from randomly chosen areas with similar population densities, the number of household members, age distribution, and collection of biological specimens from the high and low-density areas were also important factors when analyzing the data. This study was carried out to observe differences in the SAR and seroprevalence level in people living in high- and low-density areas.

We found that 10% (50/497) of contacts were SARS-CoV-2-positive as determined by RT-PCR in high-density areas, compared to 20% (37/187) in low-density areas. Studies conducted on SARS-CoV-2 transmission reported an attack rate ranging from 17 to 18.9%, which is comparable with the findings of our study conducted in low-density neighborhoods [12,13,14,15]. At the beginning of the study, it was assumed that SAR would be higher in high-density areas because of the local social structure and behavior patterns. Studies conducted on the correlation of population density and SARS-CoV-2 transmission also suggested an influence of population density. One study conducted in the United States reported that a lower population density was associated with decreased community transmission [16]. In our study, we observed that most of the high-density population lost their job or income source due to the lockdown and therefore had to migrate back to villages. Therefore, this might be one reason why the low-density population SAR was higher than that measured for the high-density population. When considering age groups, the highest levels of SARS-CoV-2 positivity were detected among contacts aged between 18 and 49 years in both high- and low-density areas (30%). This result is consistent with other reports since, in most countries, the age group between 20 and 59 years is the most numerous [9,17,18]. Males were more frequently infected, which is in contrast to what was observed in China [9]. Overall, the SAR among contacts was 13% (87/684), similar to what was observed in China and Denmark, [9,12]. We observed that the SAR was higher among people who received primary education, shared a bedroom, and earned less than 10,000 BDT (~USD 119), similar to what was found in a study conducted in Singapore, where sharing a bedroom was associated with SARS-CoV-2 transmission [17]. Basic reproduction rates were higher in high-density areas than in low-density ones. Asymptomatic contacts, who were followed up for 14 days and developed symptoms, had a similar SARS-CoV-2 positivity rate compared to other studies [12]. We found higher frequencies of seropositive participants for SARS-CoV-2 IgG antibodies in areas with a high socioeconomic level on day 1 and day 28 in comparison to areas with a low socioeconomic level. A similar study conducted in Cape Town, South Africa [18], reported a higher seroprevalence of SARS-CoV-2 antibodies in participants with a low standardized income, which is opposite to our study findings.

Moreover, participants who were working in low-income occupations and living in informal accommodations more likely tested positive for antibody responses [18]. Nearly half of Khayelitsha participants, who belonged to a partially informal township in Cape Town, were affected by overcrowding and poverty and tested positive for SARS-CoV-2 antibodies [18]. This discrepancy may be due to differences in the definition of these countries’ low and high socioeconomic status. Another limitation of the study may be linked to the small number of participants in the high socioeconomic status, which skewed the analyses. Similar disparities were observed in high-income countries like the USA. In New York City, the number of laboratory-confirmed COVID-19 cases was significantly associated with multiple socioeconomic factors, e.g., population density, dependent children, and median household income [19]. In another study, the number of laboratory-confirmed COVID-19 cases and deaths was compared to the poverty index of each USA county. It was observed that, at the beginning of the pandemic, the counties with a higher poverty index yielded a higher number of cases and deaths, and this trend was confirmed throughout the pandemic [20]. In Leicester, UK, the likelihood of testing SARS-CoV-2-positive by RT-PCR was higher in the population with a larger household and belonging to an ethnic minority [21]. Smartphone tracking data in the USA demonstrated that the ‘stay at home’ orders were less followed in low-income areas compared to high-income areas [22]. In that study, participants from low-income districts reported facing multiple physical barriers to social distancing and stay-at-home orders, which may explain the higher seroprevalence in these areas [22].

Not only antibodies levels but also the levels of SARS-CoV-2-specific memory T cells may reflect a previous infection and can be important for the establishment of a long-term immunity to COVID-19. Recently, SARS-CoV-2-specific T-cells have been identified in a subset of seronegative individuals, and, importantly, SARS-CoV-2-specific T-cells were more commonly detected in close contacts of confirmed SARS-CoV-2 patients than in blood donors [23]. Using the identification of SARS-CoV-2 RNA by PCR as a marker of infection, we may have underestimated the true prevalence of COVID-19 in our cohort in comparison to seroconversion analysis. Participants may have been infected by SARS-CoV-2, as evident by seropositivity, though remaining asymptomatic. In addition, dampened immune responses in the low-SES people may be related to the lack of T cell immune responses. Therefore, prospective seroprevalence studies in different settings (high- and low-density areas) of Dhaka city are needed to establish infection control guidelines, along with in-depth studies for measuring SARS-CoV-2-specific T-cell responses.

This study has several limitations. First, we conducted it at the beginning of the pandemic (June–September 2020). Second, we cannot claim that COVID-19-positive neighborhood contacts had not been infected by other index cases rather than by the enrolled index cases. Third, the transmission of infection could also have been possible from our defined contacts to cases. Fourth, we observed no differences related to the socio-economic conditions between high- and low-density areas among the study population for any of the variables tested. To mitigate this limitation, we further analyzed our data using the Modified Kuppuswamy Socioeconomic Scale.

## 5. Conclusions

We observed that the secondary attack rate for COVID-19 infection was higher in low-density areas. On the other hand, the basic reproduction number (R0) was higher in high-density areas in the same period. Our study shows that people with a higher socioeconomic status seroconvert significantly compared to those with a lower socioeconomic status. More in-depth studies are needed, following this cohort longitudinally and observing their nutrition patterns, behavioral practices, and household size, so to better understand the mechanism of COVID-19 infection, its nature, and its transmission process.

## Figures and Tables

**Table 1:** Distribution of the demographic characteristics of contacts in high-density and low-density areas.

Characteristic	High-Density N = 497		Low-Density N = 187	
	*n*	(%)	*n*	(%)
Median Age (range) in years	25	(0 *–95) ^ψ^	27	(3–75) ^ψ^
**Age Distribution**				
<5 years	7	(1)	1	(1)
6–10 years	33	(7)	9	(5)
11–20 years	141	(28)	42	(22)
21–30 years	120	(24)	67	(36)
31–40 years	92	(19)	24	(13)
41–50 years	60	(12)	22	(12)
51–60 years	32	(6)	13	(7)
>60 years	12	(2)	9	(5)
**Sex**				
Male	228	(46)	88	(47)
Female	269	(54)	99	(53)
**Education**				
No education	141	(28)	20	(11)
Primary	201	(40)	98	(52)
Secondary	131	(26)	49	(26)
Higher Secondary	18	(4)	10	(5)
Tertiary	6	(1)	10	(5)
**Household ⴕ**				
Household size (Median, range)	4	(1–14)	4	(1–9)
No. of bedrooms (Median, range)	1	(1–4)	2	(1–6)
Size of bedroom, sft (Median, range)	110	(12–289)	140	(13–400)
Sharing bedroom	462	(97)	136	(94)
No. of family members sharing one bedroom (Median, Range)	3	(0–7)	3	(0–12)
**Income and Expenditure ⴕ**				
Monthly income, BDT (mean, SD±)	16,942	(±12,691)	20,881	(±13,549)
Monthly expenditure, BDT(mean, SD±)	14,098	(±8284)	18,852	(±16,267)

^ψ^ Range, * 8 months, ⴕ Neighborhood contacts (N = 623).

**Table 2:** Secondary attack rate (SAR) in high-density and low-density areas in Dhaka city.

	Secondary Case				Uninfected Contacts				Secondary Attack Rate		*p-Value*
	*High*		*Low*		*High*		*Low*		*High*	*Low*	
	*n*	%	*n*	%	*n*	%	*n*	%	%	%	
**Contact type**											
Household	1	(2)	12	(32)	19	(4)	29	(19)	5	29	***<0.05***
Neighborhood	49	(98)	25	(68)	428	(96)	121	(81)	10	17	***<0.05***
Overall	50	(100)	37	(100)	447	(100)	150	(100)	10	20	***<0.05***
**Seropositivity at day 1**											
Positive	27	(54)	23	(62)	277	(62)	85	(57)	9	21	***<0.05***
Negative	23	(46)	14	(38)	170	(38)	65	(43)	12	18	*>0.05*
**Age, years**											
<18	11	(22)	6	(16)	122	(27)	25	(17)	8	19	*>0.05*
18–49	31	(62)	25	(68)	279	(62)	103	(69)	10	20	***<0.05***
≥50	8	(16)	6	(16)	46	(10)	22	(15)	15	21	*>0.05*
**Sex**											
*Male*	20	(40)	12	(32)	208	(47)	76	(51)	9	14	***<0.05***
Female	30	(60)	25	(68)	239	(53)	74	(49)	11	25	***<0.05***
**Education**											
No education	9	(18)	2	(5)	132	(30)	18	(12)	6	10	*>0.05*
Primary	26	(52)	24	(65)	175	(39)	74	(49)	13	24	***<0.05***
Secondary	14	(28)	9	(24)	117	(26)	40	(27)	11	18	*>0.05*
Higher Secondary	1	(2)	1	(3)	17	(4)	9	(6)	6	10	*>0.05*
Tertiary	0	(0)	1	(3)	6	(1)	9	(6)	0	10	*>0.05*
**Household size ⴕ**											
<6 members	43	(88)	19	(76)	332	(79)	96	(79)	11	17	*>0.05*
≥6 members	6	(12)	6	(24)	96	(23)	25	(21)	6	19	***<0.05***
**Sharing bedroom ⴕ**											
Yes	46	(94)	25	(100)	417	(97)	112	(93)	10	18	***<0.05***
No	2	(4)	0	(0)	11	(3)	9	(7)	15	0	*>0.05*
Monthly income, BDT ** ⴕ											
≤10,000	14	(29)	7	(28)	115	(27)	19	(16)	11	27	***<0.05***
>10,000	35	(71)	18	(72)	313	(73)	102	(86)	10	15	*>0.05*
Monthly expenditure, BDT ** ⴕ											
≤10,000	22	(45)	11	(44)	169	(39)	25	(21)	12	31	***<0.05***
>10,000	27	(55)	14	(56)	259	(61)	96	(79)	9	13	*>0.05*

** BDT, Bangladeshi Taka. ⴕ Neighborhood contacts (N = 623).

**Table 3:** Estimation of the basic reproduction number (Ro) in high-density and low-density areas in Dhaka city.

	Secondary Case within 14 Days		Index Case	
	*High (n = 39)*	*Low (n = 34)*	*High (n = 14)*	*Low (n = 23)*
	n	n	Basic Reproduction Number (Ro)	
**Contact type**				
Household	1	11	0.1	0.5
Neighborhood	38	23	**2.7**	1
**Age, years**				
<18	9	6	0.6	0.3
18–49	24	24	1.7	1.0
≥50	6	4	0.4	0.2
Overall	39	34	2.8	1.5
**Sex**				
Male	16	10	1.1	0.4
Female	23	24	1.6	1
**Education**				
No education	8	1	0.6	0
Primary	20	23	1.4	1
Secondary	10	8	0.7	0.3
Higher Secondary	1	1	0.1	0
Tertiary	0	1	0	0
**Household size ⴕ**				
<6 members	33	18	**2.4**	0.8
≥6 members	5	5	0.4	0.2
**Sharing bedroom ⴕ**				
Yes	35	23	**2.5**	0.3
No	3	0	0.2	0.7
Monthly income, ** ⴕ				
*≤10,000*	13	6	0.9	0.3
*>10,000*	25	17	**1.8**	0.7
Monthly expenditure, ** ⴕ				
≤10,000	19	10	1.4	0.4
>10,000	19	13	1.4	0.6

** BDT, Bangladeshi Taka. ⴕ Neighborhood contacts (N = 623).

**Table 4:** Seropositivity and level of SARS-CoV-2 antibodies in high- and low-SES people living in high- and low-density areas of Dhaka city.

	Day 1						Day 28					
	High Density			Low Density			High Density			Low Density		
	High SES (*n* = 119)	Low SES (*n* = 323)	*p* Value	High SES (*n* = 47)	Low SES (*n* = 71)	*p* Value	High SES (*n* = 119)	Low SES (*n* = 323)	*p* Value	High SES (*n* = 47)	Low SES (*n* = 71)	*p* Value
**IgG**												
^a^ Seropositivity, *n* (%)	87 (73)	192 (59)	0.011 *	31 (66)	41 (58)	0.482	88 (74)	192 (59)	0.005 **	34 (72)	43 (61)	0.237
^b^ GM (ng/mL)	827	448	0.015 *	478	460	0.783	627	365	0.029 *	525	453	0.694
**IgM**												
^a^ Seropositivity, *n* (%)	61 (51)	153 (47)	0.536	18 (38)	35 (49)	0.324	48 (40)	128 (40)	0.913	14 (30)	24 (34)	0.691
^b^ GM (ng/mL)	443	441	0.562	345	490	0.129	365	381	0.949	296	356	0.098

^a^ Statistical analysis for seropositivity in high- and low-SES groups was performed using the chi-square test. ^b^ Statistical difference in the geometric mean conc. of antibodies between high- and low-SES groups was analyzed using the Mann–Whitney U test. * *p* = <0.05; ** *p* = <0.01.

## Data Availability

Data cannot be shared publicly because they are confidential. Data are available from the respective department of icddr,b for researchers who meet the criteria for access to confidential data.

## Appendix A

### Appendix A.1. Definition of Secondary Attack Rate (SAR)

In this study, secondary attack rate (SAR) was defined as a measure of the frequency of new infections of COVID-19 among neighboring contacts of confirmed cases in a defined period and their household members (as per WHO, exposure begins 2 days prior to symptoms), determined by a positive COVID-19 result [24].

### Appendix A.2. Definition of Basic Reproduction Number (R0)

R-naught (R0) was defined as the basic reproduction number of COVID-19, the confirmed number of cases among the contacts directly generated by one case in a population where all individuals are susceptible to infection [25].

### Appendix A.3. Definition of Effective Reproduction Number (Rt)

The effective reproduction number (Rt or R) was estimated by the product of the basic reproductive number of COVID-19 and the fraction of the host population susceptible to COVID-19 infection [26].

### Appendix A.4. Definition of Serologic Response or Seroconversion

Sero-conversion can be defined as ≥two-fold increase in antibody titer in sera between enrolment and later time points (e.g., at day 1 and day 28) of neighborhood contacts [27,28,29,30,31,32].

### Appendix A.5. Operational Definition of High-Density and Low-Density Areas

High-density areas are slums which are horizontally shared spaces with more than 5 people living in a 9–12 feet by 6–8 feet room.

Low-density areas are non-slum wards, which have high-rise buildings and apartments.

**Table A1:** Response rate and levels of SARS-CoV-2 antibodies in RT-PCR-positive contacts with high-SES and low-SES in Dhaka city.

RT-PCR Positive on	SARS-CoV-2 IgG				SARS-CoV-2 IgM			
	*Day 1*		*Day 28*		*Day 1*		*Day 28*	
	High SES	Low SES	High SES	Low SES	High SES	Low SES	High SES	Low SES
**Day 1**								
Seropositivity, *n*	11/16	13/21	14/14	16/16	8/16	10/21	7/14	6/16
(%)	(69)	(62)	(100)	(100)	(50)	(48)	(50)	(38)
# GM (ng/mL)	501	695	1509	2141	528	501	485	440
**Day 7**								
Seropositivity, *n*	7/10	9/20	10/10	10/16	6/10	7/20	5/10	7/16
(%)	(70) ***	(45)	(100) ***	(63)	(60)	(35)	(50)	(44)
GM (ng/mL)	517	736	1556	2432	563	604	539	630
**Day 14**								
Seropositivity, *n*	1/3	6/15	3/3	10/11	2/3	7/15	2/3	7/11
(%)	(33)	(40)	(100) **	(91)	(67) **	(47)	(67)	(64)
GM (ng/mL)	602	313	1918	2078	476	528	509	619
**Day 28**								
Seropositivity, *n*	1/3	1/12	2/3	4/12	1/3	5/12	1/3	6/12
(%)	(33) ***	(8)	(67) ***	(33)	(33)	(42)	(33)	(50) *
GM (ng/mL)	183	61	842	150	230	396	509	490

Statistically significant differences were observed between RT-PCR-positive participants with high and low SES. Statistical analysis was performed using Fisher’s exact test. * *p* < 0.05, ** *p* < 0.01, *** *p* < 0.001. # No statistical differences were observed for Geometric mean (GM) concs. of IgG and IgM antibodies between people with high and low SES.
